# Elevated serum levels of anti-collagen type I antibodies in patients with spontaneous cervical artery dissection and ischemic stroke: a prospective multicenter study

**DOI:** 10.3389/fimmu.2024.1348430

**Published:** 2024-05-22

**Authors:** Silke Zimmermann, Markus Weißenfels, Norma Krümmer, Wolfgang Härtig, Gesa Weise, Daniela Branzan, Dominik Michalski, Johann Otto Pelz

**Affiliations:** ^1^ Institute of Laboratory Medicine, Clinical Chemistry and Molecular Diagnostics, University Hospital Leipzig, Leipzig, Germany; ^2^ Department of Neurology, Heinrich-Braun-Klinikum, Zwickau, Germany; ^3^ Department of Neurology, Klinikum Altenburger Land, Altenburg, Germany; ^4^ Paul Flechsig Institute – Center of Neuropathology and Brain Research, University of Leipzig, Leipzig, Germany; ^5^ Department of Neurology, Sana Kliniken Leipziger Land, Borna, Germany; ^6^ Department of Vascular Surgery, University Hospital Leipzig, Leipzig, Germany; ^7^ Department of Neurology, University Hospital Leipzig, Leipzig, Germany

**Keywords:** autoimmune, spontaneous cervical artery dissection (sCAD), connective tissue, anti-collagen type I antibody, vascular autoimmunity

## Abstract

**Introduction:**

Spontaneous cervical artery dissection (sCAD) is a rare vasculopathy whose trigger is still unknown. We hypothesized that autoimmunity against components of the vascular wall might play a critical role in sCAD and examined anti-collagen type I antibodies in patients with sCAD, acute ischemic stroke, patients with thromboendarterectomy, and controls.

**Methods:**

Fifty-seven patients with sCAD (age 45.7 ± 10.2 years, female 18 (31.6%)) were prospectively enrolled in four German stroke centers. Blood samples were collected at baseline, at day 10 ± 3, and after 6 ± 1 months. Patients with ischemic stroke not related to CAD (n=54, age 56.7 ± 13.7 years, female 15 (27.8%)), healthy probands (n=80, age 57.4 ± 12.9 years, female 56 (70%)), and patients undergoing thromboendarterectomy of the carotid artery (n=9, age 70.7 ± 9.3 years, female 2 (22.2%)) served as controls. Anti-collagen type I antibodies were determined by enzyme-linked immunosorbent assays (ELISAs).

**Results:**

Patients with acute sCAD had higher serum levels of anti-collagen type I antibodies (33.9 ± 24.6 µg/ml) than probands (18.5 ± 11.0 µg/ml; p <0.001) but lower levels than patients with ischemic stroke not related to sCAD (47.8 ± 28.4 µg/ml; p=0.003). In patients with sCAD, serum levels of anti-collagen type I antibodies were similar in the acute, subacute, and chronic phase. Levels of anti-collagen type I antibodies significantly correlated with circulating collagen type I (rho=0.207, p=0.003).

**Conclusion:**

Anti-collagen type I antibodies seem not to represent a trigger for acute sCAD or ischemic stroke but may rather be linked to the metabolism and turnover of collagen type I.

## Introduction

Although spontaneous cervical artery dissection (sCAD) of the internal carotid artery (ICA) or the vertebral artery (VA) is a rare vasculopathy, it is one of the most frequent causes of juvenile ischemic stroke (1, 2). While major, penetrating or blunt, cervical traumas are important predisposing risk factors for CAD, the causative role of preceding minor trauma in sCAD is less clear (2). Currently, the cause for the spontaneous dissection within the vessel wall still remains unknown in most non-traumatic cases. There is growing evidence that a subtle, in most patients clinical inapparent aberration of the vascular connective tissue may play a role in the pathophysiology of sCAD (3). However, inherited connective tissue disorders seemed exceptional even in patients with a family history of sCAD (4). A main component of the vascular extracellular matrix are collagens, in particular collagen type I and III (5). Noteworthy, sCAD shares some characteristics with the Guillain-Barré-Strohl syndrome (GBS), an acute but transient autoimmune-mediated disease of peripheral nerves following infection (6). Like in patients with GBS, those with sCAD more often suffer from preceding (airway) infections than controls (7). The recurrence of sCAD is rare and clustered around the index event, pronouncing its transient character and association with a trigger (8). Remarkably, autoimmune-mediated diseases were reported to be more prevalent in patients with sCAD (9), pointing to a susceptibility for autoimmunity.

Therefore, we hypothesized an autoimmune-mediated trigger for acute sCAD and an involvement of collagen as part of the vascular extracellular matrix. Thus, this study aimed to assess autoantibodies directed against collagen type I (anti-collagen type I antibodies) in patients with sCAD and several control groups.

## Methods

This multicenter, prospective, non-interventional, explorative study was performed according to the ethical standards laid down in the 1964 Declaration of Helsinki and its later amendments. The study was approved by the local ethics committee of the Medical Faculty at the University of Leipzig (reference number 410/18-ek). All patients or their legal representatives gave their informed and written consent to participate in this study.

### Study population

This study is part of a larger research project addressing the vascular extracellular matrix in patients with sCAD. While the findings about elastin, collagen type I, and collagen type III as biomarkers for acute sCAD were published recently (10), the present study focused on autoimmunity against collagen type I. Thus, the population of this study is the same as in Zimmermann et al. (10). Briefly, patients with sCAD were prospectively enrolled in four German stroke centers from May 2018 to June 2023. Diagnosis of sCAD of the ICA or the VA was based on typical clinical symptoms and had to be confirmed by radiological imaging with the detection of at least one of the following criteria: 1. presence of an intramural hematoma, 2. a long tapering stenosis without signs of atherosclerosis, 3. an intimal flap or a double lumen (11). Despite angiography, all patients with sCAD underwent a profound diagnostic work up including transesophageal echocardiography, an exhaustive laboratory testing including a screening for vasculitis, collagenosis, thrombophilic diathesis (anti-phospholipid-syndrome, protein C, protein S, homocystein), and a thorough assessment of the medical history. Main exclusion criteria were a known connective tissue disease and pregnancy. Due to the lack of studies that would have examined autoimmunity against components of the vascular extracellular matrix, we did not calculate a power analysis.

Patients with sCAD whose first clinical signs of the dissection occurred within 14 days before study enrollment were considered as acute (T0), while those with first symptoms >14 days before study enrollment were considered as subacute (T1). Three different populations were selected for comparisons:

- Consecutive patients with an acute ischemic stroke that was not attributed to a cervical artery dissection (non-cervical artery dissection (CAD) ischemic stroke).
- Healthy controls without known cerebro- or cardiovascular disease.
- Patients with an acute ischemic stroke due to a severe (>70% NASCET (12)), atherosclerotic stenosis of the ipsilateral ICA that underwent thromboendarterectomy.

Demographic characteristics, medical history, clinical presentation, and imaging findings of the patients and the healthy controls were recorded. Participants also completed a self-created questionnaire focusing on signs and symptoms of infections within the last six weeks before study enrollment (**Supplementary 1**).

From patients with sCAD blood samples were collected by venipuncture (serum, S-Monovette^®^, Sarstedt AG & Co, Nümbrecht, Germany) within 48 hours after hospital admission (at study enrollment, T0), at day 10 ± 3 (T1), and after 6 ± 1 months (T2). From patients with non-CAD ischemic stroke, blood samples were collected by venipuncture within 72 hours after stroke onset. Blood samples from patients undergoing thromboendarterectomy were collected by venipuncture before surgery and 7 ± 1 days after surgery. After blood sampling, tubes were kept in an upright position for 30 minutes to allow clotting, followed by a centrifugation at 5.500 g for 10 minutes. Aliquots of the supernatants were then frozen at -80°C until analysis.

### Measurements of anti-collagen type I

Serum levels of anti-collagen type I antibodies in the patients’ sera were determined by ELISAs according to the manufacturer’s instructions (Biomatik Corporation, Kitchener, Ontario, Canada). Samples were diluted according to the test range with either dilution buffer provided by the manufacturer or phosphate-buffered saline. The precision of the tests was defined via intra-assay coefficient of variation (CV, < 10%) and inter-assay coefficient of variation (< 12%). In brief, microtiter plates had been pre-coated with collagen type I. Standards and samples were then added to the appropriate plate wells followed by processing with a horseradish peroxidase-conjugated secondary antibody. After adding of tetramethylbenzidine solution, the wells containing anti-collagen I displayed color changes. The enzymatic reaction was stopped by adding sulfuric acid solution. The color changes were then measured spectrophotometrically at a wavelength of 450 nm ± 10 nm, followed by calculation of antibody concentration in each sample by interpolating from the standard curve.

### Statistical analysis

For statistical calculations, the IBM SPSS Statistics Package Version 29 (IBM Corp., Armonk, New York, USA) was used. Continuous variables were described by mean ± standard deviation, while categorical variables were expressed as counts with percentages.

Statistical significance between groups was assessed by chi square test for categorical variables, by Mann-Whitney U test for independent, or by Wilcoxon signed-rank test for dependent interval-scaled parameters. Friedman test (for independent parameters) and Kruskal-Wallis-H test (for dependent parameters) were used as global non-parametric tests. Pearson test and Spearman test were used for bivariate correlation analyzes. A p value of <0.05 was considered statistically significant.

## Results

Altogether, 72 patients with CAD were enrolled, of whom 15 (20.8%) patients had to be excluded (four patients with a traumatic CAD, seven patients with no confirmation of CAD after extensive diagnostic work-up, four patients with sCAD but symptom onset >14 days before study enrollment). This approach finally resulted in 57 patients (age 45.7 ± 10.2 years, female 18 (31.6%)) with an acute sCAD. Fifty-four patients with ischemic stroke not related to CAD (age 56.7 ± 13.7 years, female 15 (27.8%), nine patients with ischemic stroke and thromboendarterectomy of the ICA (age 70.7 ± 9.3 years, female 2 (22.2%)), and 80 healthy controls (n=80, age 57.4 ± 12.9 years, female 56 (70%)) were enrolled for control purpose.

Baseline demographics and clinical data are shown in **Table 1**. Briefly, patients with sCAD were younger (45.7 ± 10.2 years) and controls had less cardiovascular risk factors. Mean time from the onset of first sCAD-related clinical symptoms to study enrollment was 4 days.

**Table 1:** Baseline demographic data of patients with spontaneous cervical artery dissection (sCAD) and the control groups.

	Patients with acute sCAD (n = 57)	Patients with traumatic CAD (n = 4)	Patients with ischemic stroke not related to CAD (n = 54)	Patients with thromboendarterectomy (n = 9)	Controls (n = 80)	p
Age in years (mean ± SD)	45.7 ± 10.2	54.8 ± 4.3	56.7 ± 13.7	70.7 ± 9.3	57.4 ± 12.9	<0.001
Female sex (n, %)	18 (31.6%)	1 (25%)	15 (27.8%)	2 (22.2%)	56 (70%)	<0.001
NIHSS at admission (mean ± SD)	2.9 ± 4.2	6.3 ± 7.2	3.8 ± 3.6	2.0 ± 1.0	–	0.037
Time from symptom onset to study enrollment in days (mean ± SD)	4 ± 3	2 ± 1	2 ± 1	9 ± 5	–	<0.001
Location of CAD (n, %)	ICA: 38 (66.7%) VA: 19 (33.3%)	ICA: 3 (75%) VA: 1 (25%)	–	–	–	–
Multiple CAD (n, %)	3 (5.3%)	0	–	–	–	–
Arterial Hypertension (n, %)	29 (50.9%)	3 (75%)	39 (72.2%)	8 (88.9%)	29 (36.6%)	<0.001
Hyperlipidemia (n, %)	29 (50.9%)	0	40 (74.1%)	8 (88.9%)	17 (21.3%)	<0.001
Diabetes mellitus (n, %)	1 (1.8%)	1 (25%)	12 (22.2%)	2 (22.2%)	1 (1.3%)	<0.001
Current smoking (n, %)	20 (35.1%)	0	29 (53.7%)	4 (44.4%)	15 (18.8%)	<0.001

SD, standard deviation; NIHSS, National Institute of Health stroke scale.

**Figure 1:**
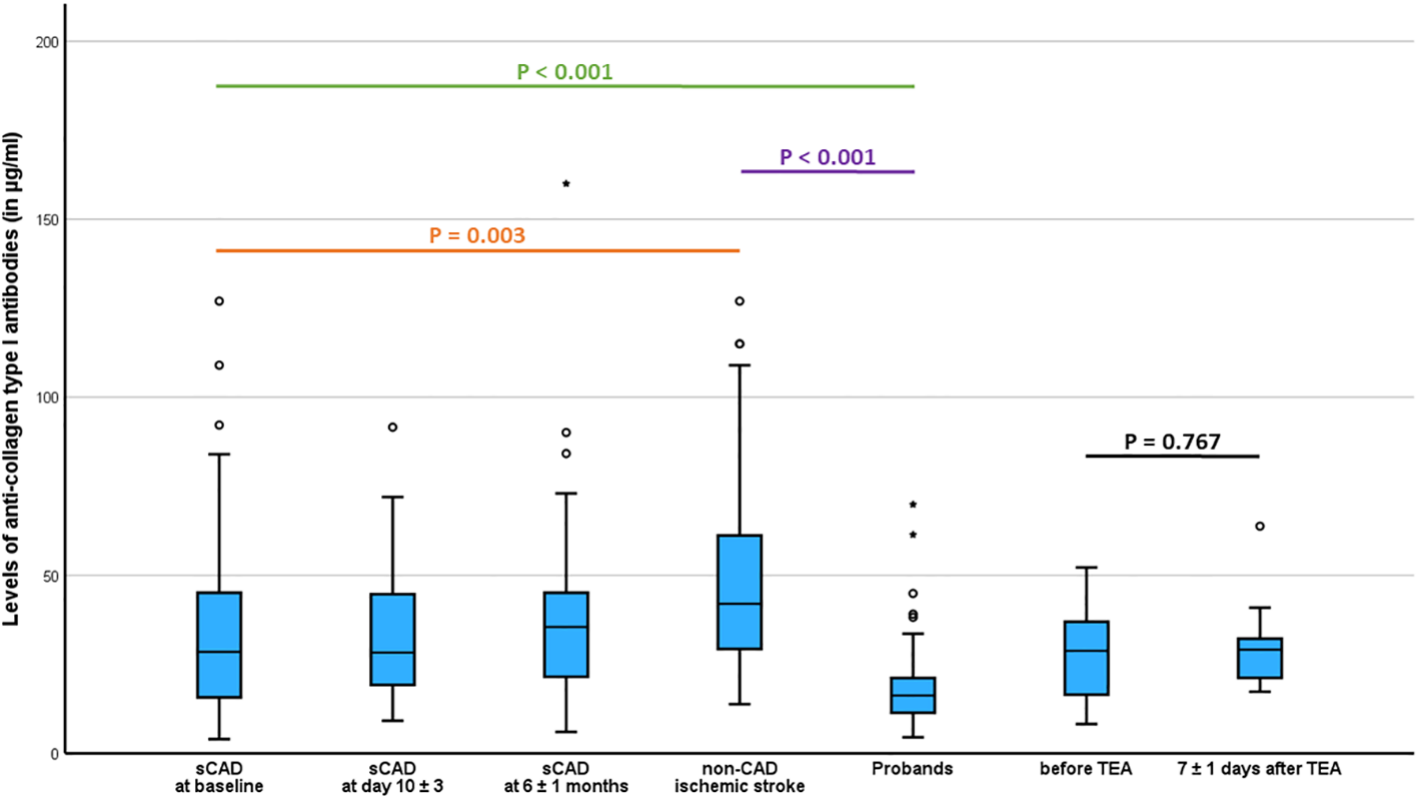
Comparison of serum levels of anti-collagen type I antibodies between patients with spontaneous cervical artery dissection (sCAD), patients with non-CAD ischemic stroke, probands, and patients with ischemic stroke undergoing thromboendarterectomy (TEA).

Patients with acute sCAD had significantly higher serum levels of anti-collagen type I antibodies than controls (33.9 ± 24.6 µg/ml *vs.* 18.5 ± 11.0 µg/ml, p < 0.001) but significantly lower levels of anti-collagen type I antibodies than patients with non-CAD ischemic stroke (33.9 ± 24.6 µg/ml *vs.* 47.8 ± 28.4 µg/ml, p = 0.003; **Table 2**, **Figure 1**). Patients with acute non-CAD ischemic stroke had significantly higher levels of circulating anti-collagen type I antibodies than controls (47.8 ± 28.4 µg/ml *vs.* 18.5 ± 11.0, p < 0.001; **Table 2**, **Figure 1**). Serum levels of anti-collagen type I antibodies were similar between patients with acute sCAD of the ICA (n=38) and the VA (n=19), respectively (33.6 ± 24.0 µg/ml *vs.* 34.5 ± 26.4 µg/ml, p = 0.966). In healthy controls levels of circulating anti-collagen type I antibodies did not differ between female and male (18.9 ± 10.3 µg/ml *vs.* 17.6 ± 12.6 µg/ml, p = 0.107), and there was no correlation between age and levels of circulating anti-collagen type I antibodies (Spearman-rho 0.103, p = 0.364). Serum levels of anti-collagen type I antibodies significantly correlated with levels of circulating collagen type I (data used for calculation, i.e. the levels of circulating collagen type I, were published recently in Zimmermann et al., 2023 (10); n = 199, Pearson-rho = 0.207, p = 0.003).

**Table 2:** Levels of anti-collagen type I antibodies in patients with spontaneous cervical artery dissection (sCAD) and control groups.

	Patients with sCAD at study enrollment (n=57)	Patients with sCAD at day 10 ± 3 (n=37)	Patients with sCAD at 6 ± 1 month (n=38)	Patients with traumatic CAD (n=4)	Controls (n=80)	Patients with ischemic stroke not related to CAD (n=54)	Patients before TEA	Patients 7 ± 1 days after TEA
anti-collagen type I in µg/ml (mean ± SD)	33.9 ± 24.6	33.5 ± 18.3	39.2 ± 28.5	33.4 ± 21.0	18.5 ± 11.0	47.8 ± 28.4	25.8 ± 15.5	30.5 ± 14.6

TEA, thromboendarterectomy; SD, standard deviation.

Within the group of patients with sCAD, levels of circulating anti-collagen type I antibodies were similar at baseline, the subacute phase (T1), and the chronic phase (T2) (33.9 ± 24.6 µg/ml *vs.* 33.5 ± 18.3 µg/ml *vs.* 39.2 ± 28.5 µg/ml, Friedman test, p = 0.852; **Table 2**). In patients with thromboendarterectomy, serum levels of anti-collagen type I antibodies were similar before and 7 days after surgery (25.8 ± 15.5 µg/ml *vs.* 30.5 ± 14.6, Wilcoxon-signed rank test, p = 0.767; **Table 2**, **Figure 1**).

Patients with acute sCAD more often reported upper airway infections within 6 weeks prior to sCAD (sCAD: 34.5%, non-CAD ischemic stroke 9.4%, controls 15%, chi square p = 0.05). However, within each group, serum levels of anti-collagen type I did not differ regarding the state of a preceding infection (**Table 3**).

**Table 3:** Levels of anti-collagen type I antibodies in patients with spontaneous cervical artery dissection (sCAD) and control groups stratified for a preceding infection.

	Patients with acute sCAD (n=55)	Patients with ischemic stroke not related to CAD (n=53)	Controls (n=80)
No infection anti-collagen type I in µg/ml (mean ± SD)	19 (34.5%) 36.0 ± 28.1	31 (58.5%) 49.2 ± 27.3	50 (62.5%) 18.5 ± 9.8
Upper airway infection anti-collagen type I in µg/ml (mean ± SD)	19 (34.5%) 37.4 ± 26.7	5 (9.4%) 37.8 ± 15.2	12 (15.0%) 16.2 ± 9.9
Other infection anti-collagen type I in µg/ml (mean ± SD)	6 (10.9%) 22.4 ± 7.1	3 (5.7%) 80.2 ± 47.3	6 (7.5%) 26.1 ± 23.0
Unclear infection anti-collagen type I in µg/ml (mean ± SD)	11 (20.0%) 27.2 ± 15.0	14 (26.4%) 43.4 ± 27.8	12 (15.0%) 17.1 ± 8.0
p (Kruskal-Wallis-H test)	0.469	0.376	0.674

Patients and controls who reported a distinct infection other than an infection of the upper airway were allocated to the “other infection” group. Participants, who reported symptoms that did not allow the clear diagnosis of an infection, were allocated to the group of “unclear infection”. The questionnaire for assessing preceding infections can be found in the supplement. TEA, thromboendarterectomy; SD, standard deviation.

## Discussion

The main finding of this study was that patients with acute sCAD had higher serum levels of anti-collagen type I antibodies than controls but lower levels than patients with ischemic stroke not related to CAD. Moreover, levels of anti-collagen type I antibodies were not associated with preceding infections.

Recently, our study group reported reduced levels of circulating elastin and collagen type III in patients with sCAD strengthening the hypothesis of an underlying, in most cases subtle and clinical inapparent, aberration of the vascular connective tissue in patients with sCAD (10). However, since an aberration of the connective tissue seems to persist over time (10, 13), it is more likely to represent a predisposition than the trigger for sCAD, which, still remains to be elucidated.

Patients with sCAD were found to have an increased susceptibility for autoimmune diseases, of whom autoimmune thyroid disease was the most frequent one (9, 14). Moreover, an increased rate of preceding infections in patients with sCAD was robustly reported (7), and markers of inflammation like peripheral leucocyte counts were elevated in patients with acute sCAD (15, 16). Notably, one study observed signs of a generalized *transient* inflammatory arteriopathy in a subset of sCAD patients that completely resolved within 6 months (17). These findings led to the hypothesis, that the transient vasculopathy in sCAD is caused by an autoantibody-mediated inflammation of the arterial wall. Thereby, collagens (in particular collagen type I and type III) are a main component of large arteries like the ICA (5, 18). Hence, we focused on autoantibodies against collagen type I and found higher levels in patients with sCAD compared to healthy controls. So far, the role of anti-collagen type I antibodies is poorly characterized. Grau and colleagues reported elevated levels of anti-collagen types I through IV antibodies in 4 of 41 (9.8%) sCAD patients (7). However, this study did not make a comparison of respective serum levels with healthy controls or patients with ischemic stroke not related to CAD. Generally, anti-collagen type I antibodies might be the consequence and not the cause of the vascular injury due to the dissection, hence, we measured anti-collagen type I antibodies before and 7 days after thromboendarterectomy and did not find any differences in patients with a defined vascular injury due to thromboendarterectomy. Since there was a positive correlation between serum levels of anti-collagen type I antibodies and levels of circulating collagen type I (10), these antibodies may play a critical role in the metabolism and turnover of collagen type I. Interestingly, patients with ischemic stroke not related to CAD had the highest levels of anti-collagen type I antibodies but also of circulating collagen type I (10). High concentrations of collagen type I were observed in fibrous atherosclerotic plaques of carotid arteries (18), and collagen type I was the predominant collagen in stable carotid plaques (19). The prevalence of carotid artery disease increases with age and is a relevant cause for ischemic stroke. On the other hand, carotid plaques are not frequently found in patients with sCAD, therewith, their increased levels of anti-collagen type I antibodies in comparison to healthy controls might rather be due to an increased metabolism and turnover of collagen type I contributing to the assumed underlying subtle vasculopathy in sCAD patients (10). A similar relationship was also reported for collagen type IV and anti-collagen type IV antibodies. Both were lower in patients with type 2 diabetes than in healthy controls, and the authors concluded that the vascular metabolism and turnover of collagen type IV within the vascular wall was decreased in patients with diabetes type 2 (20).

Despite its strength of being a prospective and multicenter study, this study also has some limitations. Since there were no former studies that addressed autoimmunity against components of the vascular extracellular matrix, we were not able to calculate a power analysis in before. Thus, we cannot exclude, that this study was underpowered to confirm the null hypothesis. However, levels of anti-collagen type I antibodies also did not differ between the three time points in patient with sCAD which makes a causative role in the pathophysiology of sCAD unlikely. Secondly, there were only four patients with traumatic CAD, we cannot provide a statistical analysis of this small group. Interestingly, like for circulating collagen type I (10), serum levels of anti-collagen type I antibodies in patients with traumatic CAD were nearly identical to the levels in those with sCAD.

## Conclusion

While addressing autoimmunity against a main component of the vascular connective tissue, autoantibodies directed against collagen type I were elevated in patients with sCAD but were even higher in patients with ischemic stroke not related to CAD. Thus, anti-collagen type I antibodies seem not to represent a trigger for acute sCAD but may rather be linked to the metabolism and turnover of collagen type I.

## Data availability statement

The raw data supporting the conclusions of this article will be made available by the authors, without undue reservation.

## Ethics statement

The studies involving humans were approved by the Medical Faculty at the University of Leipzig, Leipzig, Germany (reference number 410/18-ek). The studies were conducted in accordance with the local legislation and institutional requirements. The participants provided their written informed consent to participate in this study.

## Author contributions

SZ: Methodology, Writing – review & editing. MW: Investigation, Writing – review & editing. NK: Investigation, Writing – review & editing. WH: Writing – review & editing. GW: Investigation, Writing – review & editing. DB: Writing – review & editing. DM: Writing – review & editing. JP: Conceptualization, Formal analysis, Funding acquisition, Investigation, Project administration, Writing – original draft.
