# Supplementary material for: Elevated serum levels of anti-collagen type I antibodies in patients with spontaneous cervical artery dissection and ischemic stroke: a prospective multicenter study
Source: Front Immunol. 2024 May 22;15:1348430. doi: 10.3389/fimmu.2024.1348430 (PMC11150572; doi:10.3389/fimmu.2024.1348430)
Supplement: Supplementary file 1 [file DataSheet_1.zip › Questionaire Infection - English.DOCX]

ID of participant:

Date:

All questions refer to the *last 6 weeks*:

1. Did you have an infection within the last 6 weeks? Yes No
2. Did you have an airway infection? Yes No
3. Did you suffer from cough, common cold, sore throat, hoarseness? Yes No
4. Did you have a urinary tract infection? Yes No
5. Did you suffer from burning or painful or frequent urination? Yes No
6. Did you have an infection of the skin or the soft tissue? Yes No
7. Did you have a painful redness or swelling? Yes No
8. Did you have fever (≥38.0°C)? Yes No
9. Did you have a new headache or another new pain? Yes No
10. Did you feel tired, exhausted, or ill? Yes No
11. Did you sweat even under low exertion? Yes No
12. Did you suffer from nocturnal sweating? Yes No
13. Did you attend a general practitioner (another physician)? Yes No
14. Did you have to take antibiotics? Yes No
