# Supplementary material for: Elevated serum levels of anti-collagen type I antibodies in patients with spontaneous cervical artery dissection and ischemic stroke: a prospective multicenter study
Source: Front Immunol. 2024 May 22;15:1348430. doi: 10.3389/fimmu.2024.1348430 (PMC11150572; doi:10.3389/fimmu.2024.1348430)
Supplement: Supplementary file 1 [file DataSheet_1.zip › Questionaire Infection - German.DOCX]

Nummer des Teilnehmers:

Datum:

Alle Fragen beziehen sich auf den *Zeitraum der letzten 6 Wochen*:

1. Hatten Sie in den letzten 6 Wochen eine Infektion? Ja Nein
2. Hatten Sie einen Infekt der Atemwege? Ja Nein
3. Hatten Sie Husten, Schnupfen, Halsschmerzen, Heiserkeit etc. Ja Nein
4. Hatten Sie einen Infekt der Harnwege? Ja Nein
5. Hatten Sie Brennen, Schmerzen oder häufiges Wasserlassen? Ja Nein
6. Hatten Sie eine Infektion der Haut oder Weichteile? Ja Nein
7. Hatten Sie eine schmerzhafte Rötung oder Schwellung? Ja Nein
8. Hatten Sie Fieber (≥38,0°C)? Ja Nein
9. Hatten Sie neue (Kopf-)Schmerzen? Ja Nein
10. Haben Sie sich schlapp, abgeschlagen, matt gefühlt? Ja Nein
11. Haben Sie bereits bei leichter Belastung geschwitzt? Ja Nein
12. Hatten Sie Nachtschweiß? Ja Nein
13. Waren Sie beim Hausarzt (anderem Arzt)? Ja Nein
14. Mussten Sie ein Antibiotikum eingenommen? Ja Nein
